# Postoperative Outcomes in Surgical Endodontic Treatment Using Various Root-End Filling Materials for Immature Root Apices

**DOI:** 10.7759/cureus.80801

**Published:** 2025-03-18

**Authors:** Sweta Rastogi, Shahnaz Nabi, Sanjay Miglani, Mohd I Ansari, Ajinkya M Pawar

**Affiliations:** 1 Conservative Dentistry and Endodontics, Faculty of Dentistry, Jamia Millia Islamia, New Delhi, IND; 2 Conservative Dentistry and Endodontics, Nair Hospital Dental College, Mumbai, IND

**Keywords:** apexification, cone-beam computed tomography (cbct), immature root apex, periapical surgery, root canal therapy

## Abstract

The present case series describes the postoperative outcomes of surgical endodontic treatment in teeth with immature root apices using different root-end filling materials. Three cases involving young patients with discolored and non-vital teeth were treated using varied approaches: no root-end filling, mineral trioxide aggregate (MTA), and Biodentine (Septodont, Saint-Maur-des-Fossés, France). Each case included comprehensive pre-surgical assessments, conservative non-surgical root canal treatments, and periapical surgeries performed under magnification. The outcomes were evaluated based on clinical and radiographic success over follow-up periods ranging from six to 24 months. Results demonstrated favorable healing and functional restoration across all cases, highlighting the importance of individualized treatment plans, meticulous surgical techniques, and rigorous postoperative care. The findings underscore the efficacy of contemporary root-end filling materials like MTA and Biodentine in promoting periapical healing and bone regeneration while also emphasizing the necessity for personalized approaches in managing immature root apices. Further research is recommended to optimize these treatment protocols.

## Introduction

Dental caries and dental trauma are prevalent issues that can significantly impact the integrity of teeth during their development [1]. When these conditions cause the dental pulp to become non-vital before the root has fully matured and the apical closure is complete, normal root development is arrested. This results in roots with extremely thin canal walls and wide-open apical foramina, which pose considerable challenges for root canal instrumentation and obturation, ultimately hindering the formation of a proper apical stop [2].

To address these challenges, clinicians employ apexification - a process defined by the American Association of Endodontists (AAE) as the induction of a calcified barrier in a root with an open apex or the continued apical development of an incompletely formed root in teeth with necrotic pulp [3]. Various methods exist for achieving root-end barrier formation, including multi-visit apexification using calcium hydroxide, orthograde one-visit apexification using mineral trioxide aggregate (MTA) and Biodentine (Septodont, Saint-Maur-des-Fossés, France), revascularization techniques, and periapical surgery with root-end filling [4].

One-visit apexification, as described by Mohammadi, involves the non-surgical condensation of a biocompatible material into the apical end of the root canal to create an immediate apical stop, allowing for prompt root canal filling [5]. The critical aspect of managing teeth with open apices is achieving a reliable apical seal. Over the years, various materials such as amalgam, glass ionomer cement (GIC), SuperEBA (Keystone Industries, Gibbstown, NJ), MTA, and Biodentine have been utilized to enhance apical sealing during periapical surgery [6].

It has been demonstrated that MTA is a very successful root-filling material for sealing immature root canals with open apices, making it a suitable substitute material for the apexification process [7]. Because of its excellent canal sealing qualities and biocompatibility, it is also utilized as a root end-filling and perforation repair material. It promotes periodontal ligament repair as well as the development of cementum and dentinal bridges. It actively encourages the creation of hard tissue and enhances periradicular healing because it can increase the release of cytokines from bone cells [8]. Biodentine, a novel class of dental material created by Gilles and Olivier, was introduced in 2010, which combined superior biocompatibility, good mechanical qualities, and bioactive behavior [9,10]. Biodentine offers similar properties to those of MTA, with better consistency and faster setting time [11].

The present case series aims to focus on the clinical and radiographic outcomes of different root-end filling materials used in surgical endodontic treatments for teeth with immature apices. By comparing these outcomes, this study guides us regarding a myriad of effective materials and techniques to ensure successful postoperative results, thereby improving the prognosis for teeth with challenging anatomical conditions.

## Case presentation

The series involves three cases of teeth with incompletely formed apices and blunderbuss canals treated by endodontic surgery. Each case utilized a different method to achieve an apical seal. A standardized case history was recorded, and comprehensive clinical and radiographic examinations were conducted, including cone beam computed tomography (CBCT) (KODAK 9000 3D system, Carestream Health Ltd., Rochester, NY) to precisely assess the size of the lesions. Medical histories were reviewed to guide treatment planning. All the patients were given postoperative instructions and a prescription for antibiotics and analgesics following the surgical procedure. They were evaluated 24 hours post-surgery, and sutures were removed after one week. Review appointments were scheduled at three, six, 12, and 24 months to assess bone healing.

Common preliminary procedures

Written informed consent was obtained from each patient, with thorough explanations of the associated risks and benefits of the procedures. Pre-surgical assessments were performed to ensure the patients' suitability for surgery and to prepare for the optimal surgical approach. Initial treatment involved non-surgical root canal therapy, followed by the planning of endodontic surgery. All surgical procedures were performed under magnification using loupes (Admetec, Haifa, Israel, ×3.2). After disinfecting the extraoral surface, teeth, and mucosal surfaces with betadine (Belco Pharma, Bahadurgarh, India), local anesthesia was administered using infiltration and an anterior superior alveolar nerve block using lidocaine 2% with 1:80,000 epinephrine (Lignox A, Warren Pharmaceuticals Ltd., Mumbai, India). Two vertical releasing incisions and a sulcular incision were made, and a full-thickness trapezoidal mucoperiosteal flap was reflected. The exposed tissues were kept moist with sterile saline throughout the surgery to prevent dehydration of the bone and soft tissues. After flap reflection, the lesion was localized using a probe. Osteotomy was performed using a round tungsten carbide bur (SS White Carbide Burs, SS White Dental, Lakewood, NJ) in a straight handpiece under continuous saline and betadine irrigation to access up to 3 mm of the root apex. Complete curettage was done using Hu-Friedy curettes (Hu-Friedy, Chicago, IL) to ensure the removal of all granulation tissue and achieve a clear field. Hemostasis was obtained using Gelfoam (Pharmacia & Upjohn Company LLC, Kalamazoo, MI).

Case I

A 19-year-old female patient presented to the Department of Conservative Dentistry with a chief complaint of discolored and fractured tooth in the upper front region. The patient reported a history of trauma at the age of nine years and previous dental treatment initiated at a private clinic. Clinical examination revealed a discolored maxillary left central incisor (#21) with an Ellis class III fracture but no mobility. Initial radiographic examination showed that #21 had a radiopaque material inside the root canal, an incompletely formed apex, and a blunderbuss canal configuration.

The initial treatment plan involved conservative non-surgical root canal therapy. After obtaining written informed consent, the material filling the root canal, identified as Metapex, was removed. The patient then underwent non-surgical multi-visit calcium hydroxide treatment over one year, with monthly radiographs taken to monitor the formation of an apical stop.

However, during treatment, the patient developed an intraoral swelling, and the crown of the tooth fractured at the cementoenamel junction (CEJ). Cone beam computed tomography (CBCT) revealed periapical radiolucency, external root resorption, and an incompletely formed apex in #21, indicating a guarded prognosis. The patient was presented with two treatment options: endodontic surgery or extraction followed by replacement with an implant. The patient opted against extraction due to some financial constraints and an inclination to save the natural tooth, leading to the decision to proceed with periapical surgery.

**Figure 1:**
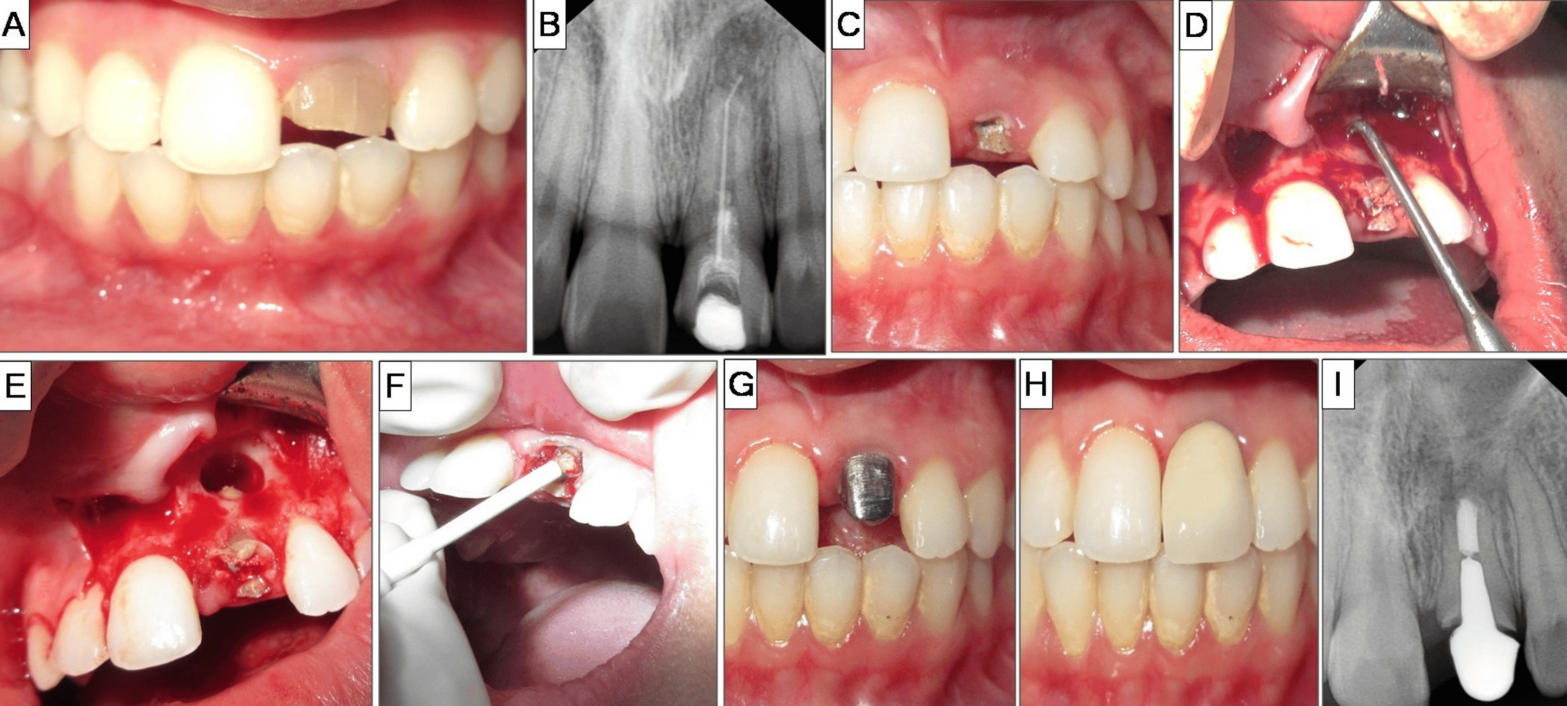
Case I: no root-end filling A) Preoperative photograph showcasing discolored and fractured tooth #21. B) Preoperative radiograph suggestive of incomplete endodontic treatment in tooth #21. C) Crown fracture at cemento-enamel junction of tooth #21. D) Lesion localization and curettage. E) Lesion area post-curettage. F) Cauterization procedure. G) Cemented cast post in tooth #21. H) Porcelain-fused-to-metal crown placed in #21. I) Two-year follow-up radiograph.

Root-end resection was performed under continuous saline and betadine irrigation using a straight fissured tungsten carbide bur. The resected root surface was examined. For this case, no root-end preparation or filling was done. The location of the root apex was estimated, and the gutta-percha was sealed at the apex with a hot burnisher. The decision against root-end preparation and filling was due to the crown fracturing at the CEJ, jeopardizing the crown-root ratio and affecting its restorability. The flap was repositioned and sutured with 5-0 Mersilk (Ethicon, Johnson & Johnson, New Brunswick, NJ) using multiple interrupted sutures, which were removed after seven days. After six months, the patient was asymptomatic, and intraoral periapical radiograph (IOPA) radiograph showed appreciable healing in the periapical region. A post-endodontic restoration was planned, involving a post and core. Since the crown of #21 was fractured at the CEJ, cauterization was performed to expose the tooth surface for placing the crown margin. Post-space preparation was done, and a wax pattern was prepared. The cast post was cemented using zinc phosphate cement (Ammdent, Punjab, India). An impression was made using the putty-wash technique, and a porcelain-fused-to-metal crown was cemented using GIC (GC Gold Label 1, GC Corporation, Tokyo, Japan). Radiographic assessment at one year showed complete healing of the surgical site. The periapical index (PAI) score used to assess the periapical health with respect to #21 improved from three to one. The overall management of case I is depicted in Figure 1.

Case II

A 23-year-old female patient presented to the Department of Conservative Dentistry with a chief complaint of discolored teeth in the upper front region. She reported a history of restorative dental treatment in her childhood due to decay. Clinical examination revealed that both the maxillary central incisors (#11 and #21) were discolored and restored with composite, with no mobility. Radiographic examination showed incompletely formed apices with blunderbuss canal configurations, shortened roots, and periapical radiolucency in both incisors.

The treatment plan involved conservative non-surgical root canal treatment with multi-visit calcium hydroxide therapy in right maxillary central incisor (#11) and without calcium hydroxide therapy in left maxillary central incisor (#21). A written informed consent was obtained.

Non-surgical root canal treatment was initiated on the same day for both teeth. After access opening under rubber dam isolation (Coltène/Whaledent, Langenau, Germany), copious irrigation with sodium hypochlorite (Hyposol, Prevest DenPro Ltd, Jammu, India) and saline was performed. After a few days, biomechanical preparation was completed, and calcium hydroxide dressing (Avuecal, Dental Avenue, Mumbai, India) was placed in #11. No dressing was placed in #21, which served as a comparator. The calcium hydroxide dressing in #11 was replaced every one to three months based on radiographic evaluation, while #21 was kept under observation.

Over six months, the patient developed intermittent intraoral swelling related to #21. Clinical examination revealed an apical stop in #11, which was not discernible radiographically. CBCT showed periapical radiolucency and buccal cortical plate perforation in relation to #21, along with incompletely formed apices in both teeth. Due to the complexity of the case, manifested as symptomatic recurrent swelling and the patient's preference to avoid further non-surgical treatment, periapical surgery was planned.

**Figure 2:**
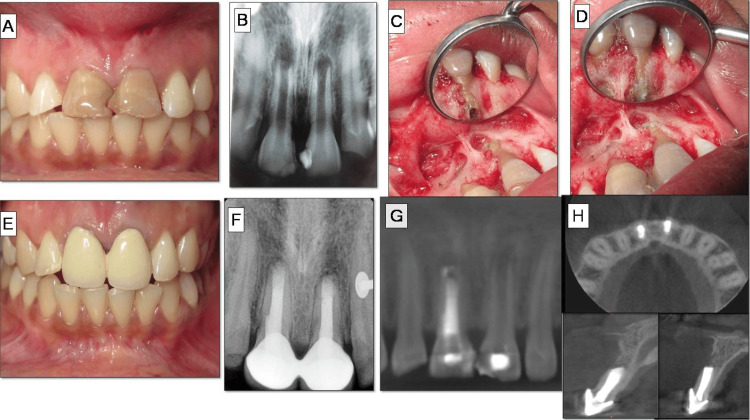
Case II: root-end filling with mineral trioxide aggregate (MTA) A) Preoperative photograph suggestive of discoloration and faulty restoration in tooth #11, #21. B) Preoperative radiographs revealing open apex with periapical lesions. C) Lesion localization and curettage. D) Root-end preparation. E) MTA root-end filling. F) Crown cementation. G) One-year follow-up radiograph. H) Preoperative cone beam computed tomography (CBCT) scan revealing wide open apices in a panoramic view of #11, #21.

Root-end resection was performed under continuous saline and betadine irrigation using a straight fissured tungsten carbide bur. The resected root surface was inspected. Root-end preparation was done using Ultrasonic Pro Ultra Surgical Endo Tips, SURG 1 and 2 (Dentsply Tulsa Dental Specialties, Johnson City, TN) up to 3 mm into the root apex, followed by irrigation, drying, and isolation. Root-end filling was done with MTA (Pro Root MTA, Dentsply Sirona, York, PA). Radiographs were taken during the procedure to confirm the root-end filling. The flap was approximated back and sutured using 5-0 Mersilk with multiple interrupted sutures, which were removed after seven days. After six months, recall radiographs showed appreciable bone healing, and the patient was asymptomatic. PAI showed remarkable healing as the score progressed from four to one in #11 and from three to one in # 21, respectively. Definitive restorations included porcelain-fused-to-metal (PFM) crowns for both the maxillary central incisors. The overall management of case II is depicted in Figure 2.

Case III

A 26-year-old male presented to the Department of Conservative Dentistry with a chief complaint of discolored teeth in the upper front region. The patient reported a history of childhood trauma. Clinical examination revealed discoloration of the maxillary right central incisor (#11) and intraoral swelling in the mid-palatine raphe region. Thermal and electric pulp tests showed no response in the maxillary central incisors (#11, #21) and right lateral incisor (#12). Radiographic examination revealed incompletely formed apices with blunderbuss canal configurations, shortened roots, and periapical radiolucency associated with the maxillary right central incisor. The treatment plan included conservative non-surgical root canal therapy for all three teeth, with regenerative endodontic treatment for #11.

Non-surgical root canal treatment was initiated under rubber dam isolation. Biomechanical preparation was performed, and calcium hydroxide dressing was placed in #11, #12, and #21. After one week, the triple antibiotic paste was placed in the root canal of #11, and the patient was kept under observation for two weeks. Despite the treatment, the patient developed an intraoral palatal swelling. CBCT (KODAK 9000 3D system, Carestream Health Ltd., NY, USA) revealed periapical radiolucency with respect to #11 and perforation of the palatal cortical plate in the apical region. Due to the lack of appropriate response to regenerative endodontic therapy attempted in #11 and financial constraints, periapical surgery was planned for further management of both the central and lateral incisors.

Root-end resection was performed under continuous saline and betadine irrigation using a straight fissured tungsten carbide bur. The resected root surface was inspected. Root-end preparation was done using ultrasonic Ultrasonic Pro Ultra Surgical Endo Tips, SURG 1 and 2, up to 3 mm into the root apex, followed by irrigation, drying, and isolation. Root-end filling was performed with Biodentine, and a demineralized freeze-dried bone allograft (DFDBA) (Tata Memorial Hospital, Mumbai, India) mixed with saline was placed in the bony cavity.

**Figure 3:**
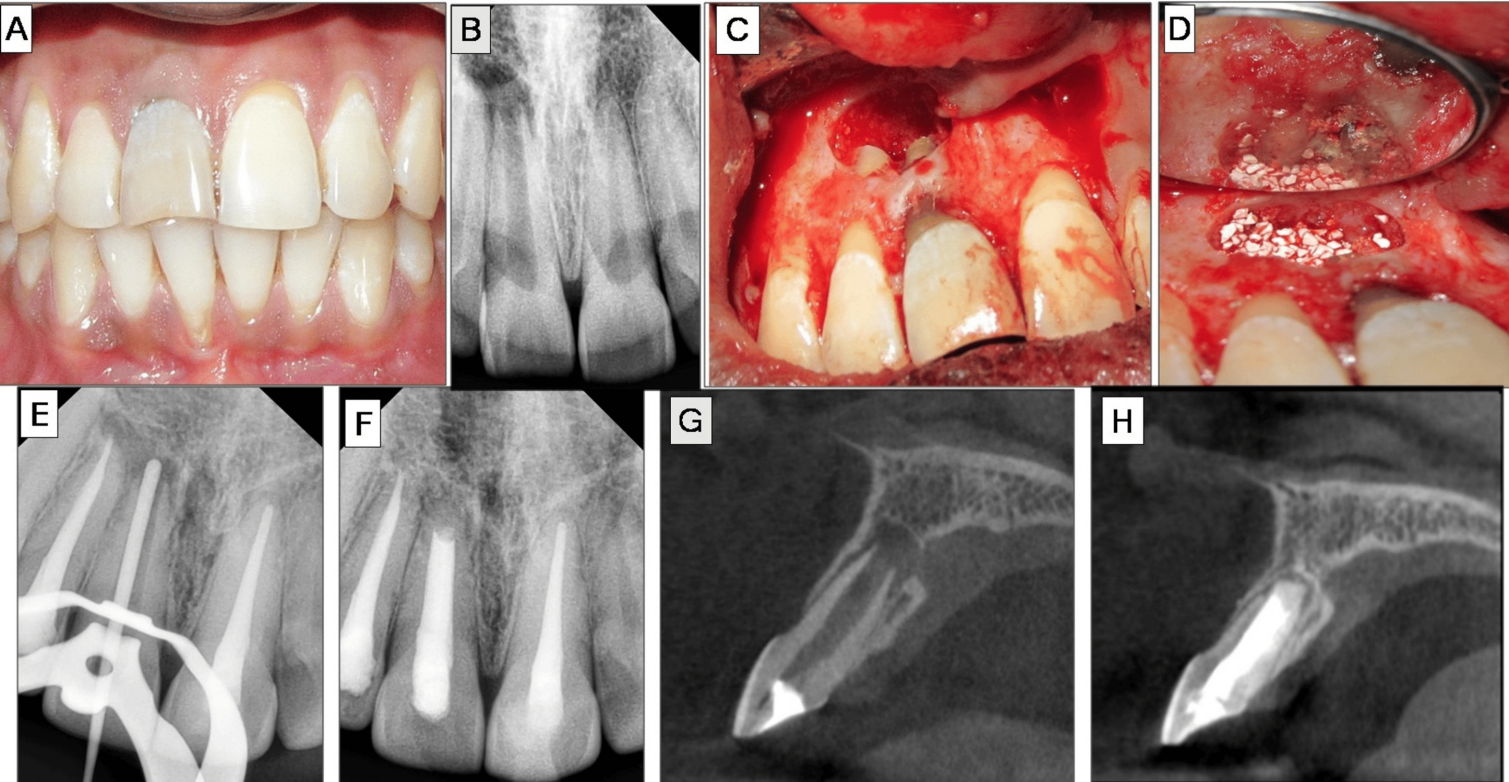
Case III: root-end filling with Biodentine A) Non-vital maxillary central incisor (#11). B) Preoperative radiographs revealing open apex with a periapical lesion in #11. C) Lesion localization and curettage. D) Root-end filling with Biodentine and bone graft placement. E) Master-cone radiograph #11. F) One-year follow-up radiograph. G) Preoperative cone beam computed tomography (CBCT) scan showcasing open apex and denudation of palatal cortical plate in cross-section view of #11. H) CBCT scan of #11.

Radiographs were taken during the procedure to confirm the root-end filling. The flap was approximated and sutured with 5-0 Mersilk using multiple interrupted sutures, which were removed after seven days. At the one-month recall, there was no intraoral swelling. A one-year recall radiograph showed appreciable bone healing, and the patient was asymptomatic. Besides, the PAI also represented a significant improvement as the scores reverted back from four to one for #11. Non-vital bleaching was planned for #11. The overall management of case III is depicted in Figure 3.

## Discussion

The case series presents a comprehensive analysis of surgical endodontic treatment outcomes in teeth with immature apices using different root-end filling materials. Each case was meticulously documented to highlight the unique challenges and clinical decisions involved in managing immature root apices.

The first case demonstrated the approach of not using a root-end filling material due to compromised restorability. The decision to seal the apex with a hot burnisher was driven by the need to maintain as much structural integrity as possible, given the fracture at the level of the CEJ [12]. Despite not using a root-end filling, the case showed significant healing and absence of symptoms, suggesting that, in specific situations, conservative approaches can still yield positive outcomes.

The second case utilized MTA, which is a material known for its excellent sealing properties and biocompatibility. MTA has been extensively studied and is often preferred for its ability to promote periapical healing and induce hard tissue formation at the root end [13]. Follow-up radiographs of the case confirmed the efficacy of MTA in achieving a reliable apical seal and promoting significant bone healing, aligning with findings from various studies that highlight its superior performance in endodontic surgeries [14,15].

The third case involved the use of Biodentine, a relatively newer material compared to MTA, known for its biocompatibility, bioactivity, and ability to promote dentin regeneration [16]. The successful outcome in this case, characterized by appreciable bone healing and absence of symptoms, supports the growing body of evidence favoring Biodentine as an effective alternative to MTA for root-end fillings. Its ease of handling and shorter setting time also make it a valuable option in clinical practice [17,18].

The cases collectively underscore the importance of individualized treatment planning in endodontic surgery. The decision-making process was guided by a combination of clinical findings, radiographic evidence, patient symptoms, and material properties. The use of CBCT in these cases provided critical insights into the extent of periapical lesions and anatomical challenges, facilitating more precise surgical interventions. The favorable outcomes across all cases highlight the efficacy of modern root-end filling materials in promoting healing and functional restoration of teeth with immature apices.

PAI has clearly demonstrated the improved postoperative outcome obtained in all three cases, respectively. Ørstavik et al. introduced the PAI scoring system to qualitatively analyze periapical health in cases of apical periodontitis in epidemiological studies, clinical trials, and clinical practice for assessing endodontic outcomes [19]. PAI index, as illustrated in Table 1, consists of five scores wherein scores 1 and 2 represent success/a healthy state and scores ranging from three to five represent failure/diseased state [20].

**Table 1:** Periapical index scoring criteria

Scores	Scoring criteria
1	Normal periapical anatomy
2	Mild changes in bone pattern
3	Changes in bone pattern with diffuse loss of minerals
4	Apical periodontitis with definite radiolucency in the periapical area
5	Severe periodontitis with features of exacerbation

However, the variability in the duration of healing and the occurrence of intermittent symptoms in some cases emphasize the need for rigorous postoperative monitoring and follow-up.

Future research should aim to further refine these treatment protocols, potentially incorporating advanced imaging techniques and novel biomaterials to enhance predictability and long-term success rates. Comparative studies with larger sample sizes and longer follow-up periods would provide more robust evidence to guide clinical practice. Overall, this case series contributes valuable insights into the management of immature root apices, demonstrating that both MTA and Biodentine are effective in achieving favorable clinical and radiographic outcomes. The choice of material and technique should be tailored to the specific clinical scenario, ensuring optimal patient care and treatment success.

## Conclusions

The present case series underscores the importance of carefully planned management strategies required for treating immature root apices using diverse root-end filling materials. Each case, whether opting for no root-end filling, MTA, or Biodentine, demonstrated unique challenges and successes. The first case highlighted the necessity for careful surgical planning despite foregoing root-end filling due to compromised restorability, achieving satisfactory healing outcomes. The second case illustrated the efficacy of MTA in providing a reliable apical seal and promoting significant periapical healing. The third case showcased the effectiveness of Biodentine in securing apical integrity and fostering bone regeneration. These findings emphasize the importance of tailored treatment plans, meticulous surgical technique under magnification, and diligent postoperative care in achieving favorable outcomes in endodontic surgery. Further research is warranted to refine these approaches and enhance their predictability and long-term success rates.
